# 3D-Printed Collagen–Nanocellulose Hybrid Bioscaffolds with Tailored Properties for Tissue Engineering Applications

**DOI:** 10.1021/acsabm.3c00767

**Published:** 2023-12-05

**Authors:** Andreja Dobaj Štiglic, Florian Lackner, Chandran Nagaraj, Marco Beaumont, Matej Bračič, Isabel Duarte, Veno Kononenko, Damjana Drobne, Balaraman Madhan, Matjaž Finšgar, Rupert Kargl, Karin Stana Kleinschek, Tamilselvan Mohan

**Affiliations:** † Faculty of Mechanical Engineering, Laboratory for Characterization and Processing of Polymers, University of Maribor, Smetanova ulica 17, 2000 Maribor, Slovenia; ‡ Institute of Chemistry and Technology of Biobased System (IBioSys), Graz University of Technology, Stremayrgasse 9, 8010 Graz, Austria; § Ludwig Boltzmann Institute for Lung Vascular Research, Stiftingtalstrasse 24, 8010 Graz, Austria; ∥ Department of Chemistry, Institute of Chemistry o Renewable Resources, University of Natural Resources and Life Sciences Vienna (BOKU), A-3430 Tulln, Austria; ⊥ Department of Mechanical Engineering, Centre for Mechanical Technology and Automation (TEMA), Intelligent Systems Associate Laboratory (LASI), University of Aveiro, 3810-193 Aveiro, Portugal; # Department of Biology, Biotechnical Faculty, Večna pot 111, 1000 Ljubljana, Slovenia; ∇ CSIR-Central Leather Research Institute, Chennai 600 020, Tamil Nadu, India; ○ Faculty of Chemistry and Chemical Engineering, Laboratory for Analytical Chemistry and Industrial Analysis, University of Maribor, Smetanova ulica 17, 2000 Maribor, Slovenia; ◆ Institute of Automation, Faculty of Electrical Engineering and Computer Science, University of Maribor, Koroska cesta 46, 2000 Maribor, Slovenia

**Keywords:** nanofibrillated cellulose, carboxymethyl cellulose, collagen, citric acid, 3D printing, cross-linking, hybrid scaffolds

## Abstract

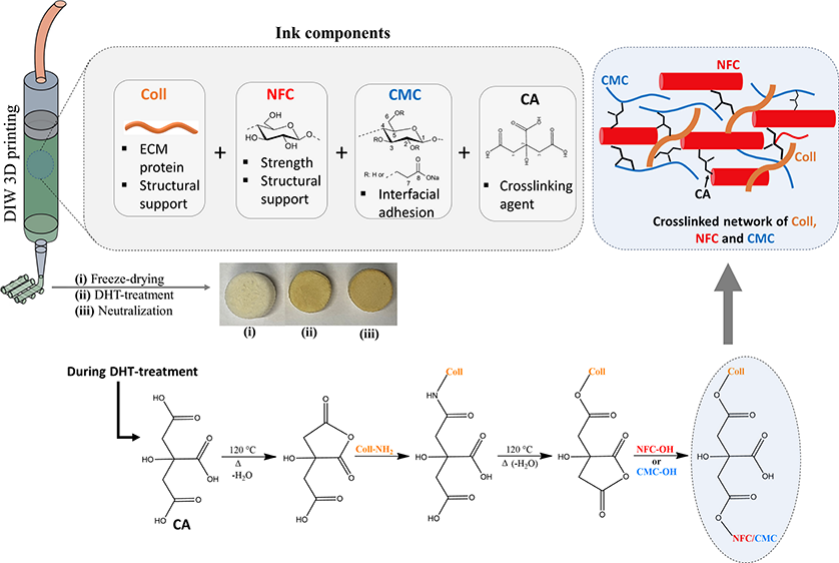

Hybrid collagen (Coll) bioscaffolds have emerged as a promising solution for tissue engineering (TE) and regenerative medicine. These innovative bioscaffolds combine the beneficial properties of Coll, an important structural protein of the extracellular matrix, with various other biomaterials to create platforms for long-term cell growth and tissue formation. The integration or cross-linking of Coll with other biomaterials increases mechanical strength and stability and introduces tailored biochemical and physical factors that mimic the natural tissue microenvironment. This work reports on the fabrication of chemically cross-linked hybrid bioscaffolds with enhanced properties from the combination of Coll, nanofibrillated cellulose (NFC), carboxymethylcellulose (CMC), and citric acid (CA). The bioscaffolds were prepared by 3D printing ink containing Coll-NFC-CMC-CA followed by freeze-drying, dehydrothermal treatment, and neutralization. Cross-linking through the formation of ester bonds between the polymers and CA in the bioscaffolds was achieved by exposing the bioscaffolds to elevated temperatures in the dry state. The morphology, pores/porosity, chemical composition, structure, thermal behavior, swelling, degradation, and mechanical properties of the bioscaffolds in the dry and wet states were investigated as a function of Coll concentration. The bioscaffolds showed no cytotoxicity to MG-63 human bone osteosarcoma cells as tested by different assays measuring different end points. Overall, the presented hybrid Coll bioscaffolds offer a unique combination of biocompatibility, stability, and structural support, making them valuable tools for TE.

## 1 Introduction

In tissue engineering (TE), developing three-dimensional (3D) porous bioscaffolds with appropriate mechanical and biological properties is critical for tissue regeneration and repair.^1^ Recent advances in 3D printing have opened up new possibilities for the fabrication of complex and customized bioscaffolds whose structure, composition, porosity, mechanical properties, etc. can be precisely controlled.^2^ Over the years, researchers have explored various biomaterials to develop 3D-printed bioscaffolds that mimic the extracellular matrix (ECM) and provide suitable properties for TE.^3^ Among the biomaterials of interest, collagen (Coll) has been widely used as a scaffold in various TE applications (e.g., skin, bone, cartilage, cardiovascular and neural tissue regeneration) as it offers good biocompatibility, biodegradability, and an environment very similar to natural ECM.^4,5^ However, they also have some disadvantages, such as limited mechanical strength, rapid degradation when used in a complex biological environment (wet or hydrated state), and possible contamination (e.g., immunogenicity), etc.^5,6^ To overcome this, Coll can be cross-linked or hybridized with polysaccharide materials to improve the overall performance of the bioscaffolds. The combination of Coll and polysaccharides offers a synergistic approach in TE, as Coll-polysaccharide hybrid bioscaffolds can be engineered to have the desired morphology, porosity, swelling, degradation, and mechanical properties such as stiffness and elasticity in the wet state, which are critical for various tissue types.^7,8^ Among other polysaccharides, nanofibrillated cellulose (NFC) and carboxymethylcellulose (CMC) have received much attention due to their unique properties and potential applications in TE.^9,10^ NFC, which is derived from renewable sources such as plant cell walls, has remarkable mechanical properties, including high strength and stiffness, making it suitable for load-bearing applications. In addition, NFC has a large surface area and unique surface chemistry that enables modification with new functional molecules.^11^ On the other hand, CMC is water-soluble, structurally similar to NFC, and has an intrinsic affinity to NFC through interfacial adhesion. Therefore, it can impart flexibility, strength, and dimensional stability to printed bioscaffolds, similar to what has been observed with alginate.^12,13^

Direct-ink-writing (DIW) 3D printing, a particular extrusion-based technique, enables the controlled deposition of inks to create multifunctional bioscaffolds from various natural or synthetic hydrogels.^10,14,15^ However, DIW of Coll alone can be challenging regarding viscosity, layer adhesion, structural integrity, shape fidelity, and mechanical stability.^16,17^ As mentioned above, these issues can be addressed by developing Coll-NFC-CMC hybrid bioscaffolds that leverage the strengths of the three materials. Furthermore, DIW printing enables the fabrication of scaffolds with well-defined, controlled, consistent structures in terms of their internal architecture, external geometry, strand size, and pore size and distribution.^18^ Such printed Coll-NFC-CMC hybrid bioscaffolds also offer good bioactivity, creating a biocompatible environment for in vitro cell or tissue growth. Multistage chemical cross-linking methods have often been used to improve the mechanical and dimensional stability of printed bioscaffolds.^19^ However, chemical cross-linkers are often organic or require pretreatment with reactive functional groups that are associated with cytotoxicity and require extensive purification.^20^ In this work, our motivation was to fabricate 3D-printed and freeze-dried Coll-NFC-CMC hybrid bioscaffolds that were cross-linked with citric acid (CA), a nontoxic cross-linker. Cross-linking was achieved by dehydrothermal (DHT) treatment in the dry state. This solvent-free treatment is commonly used to cross-link and improve the mechanical properties of Coll molecules.^21^ Although there are reports on the use of CA to cross-link cellulose-based materials,^22−24^ to our knowledge, no studies have been reported on the cross-linking of Coll-NFC-CMC hybrid bioscaffolds with CA by DHT treatment^25^ and their use for the growth of human bone osteosarcoma cells (MG-63). The novelty of this work is laid on the fabrication of Coll-NFC-CMC hybrid bioscaffolds with adequate interconnected pores or porosity, biocompatibility, and long-term dimensional and mechanical stability in a complex biological environment (e.g., cell growth medium or biofluid). All of these properties are important for long-term and successful cell growth. In addition, the biocompatibility of MG-63 cells with our hybrid bioscaffolds was tested by using different cell assays to ensure the suitability of the scaffolds for various TE applications.

In this study, we investigated for the first time the fabrication of mechanically stronger and porous Coll-NFC-CMC hybrid bioscaffolds by combining DIW 3D printing, freeze-drying, and DHT-assisted chemical (solvent-free) treatment. 3D-printed and freeze-dried Coll-NFC-CMC hybrid bioscaffolds containing different amounts of Coll were cross-linked with CA at elevated temperatures. We investigated the influence of the Coll concentration and its effects on the performance of the bioscaffolds. Therefore, the neutralized bioscaffolds were analyzed in terms of their morphology, pores, composition, structure, thermal behavior, swelling capacity, degradation, and mechanical strength in both dry and hydrated states. The safety of the cross-linked bioscaffolds in TE was evaluated using the viability of MG-63.

## 2 Experimental Section

### 2.1 Materials

The sodium salt of CMC (DS_COOH_ = 0.9, *M*_wt_ = 700 kDa), phosphate-buffered saline (PBS) (Bioperformace certified, pH 7.4), streptomycin, and penicillin were purchased from Sigma-Aldrich (Graz, Austria). NFC (3 wt % solid content) was purchased from the University of Maine, Process Development Center, USA. CA (≥99.5%) was purchased from Carl-Roth, Austria. According to the published protocol, Coll type I was isolated from the bovine Achilles tendon (see the Supporting Information).^26^ Advanced Dulbecco’s Modified Eagle’s Medium (ADMEM) and fetal bovine serum (FBS) were purchased from ThermoFisher, Germany. Ultrapure water (Milli-Q System, Millipore, USA; resistivity >18.18 M Ω cm) was used to prepare all samples.

### 2.2 Ink Development for DIW Printing

The following procedure was used to prepare Coll-NFC-CMC-CA hybrid inks for DIW printing. Initially, the freeze-dried Coll flakes (0.1, 0.5, or 1 g) were dissolved in 10 g (6.03 mL) of CA solution. To achieve complete dissolution of Coll, the solution was stirred with a mechanical laboratory stirrer (IKA EUROSTAR 20) at 200 rpm and heated at 37 °C in an oil bath for up to 3 days. To this Coll, solution (44 g), 6 g of CMC powder was slowly added and stirred with a mechanical laboratory stirrer for 30 min at 150–350 rpm. After achieving a complete dissolution of CMC powder in Coll solution, 50 g of NFC sample (3 wt % solids, as received according to the manufacturer, see section 2.1) was added and stirred with a mechanical stirrer at 200 rpm until no more NFC fibers were visible (about 20 min). The finished mixture (referred to as inks) was covered with aluminum foil and stored in the refrigerator at 2–8 °C until further use. All inks (see Table 1) were equilibrated to room temperature before DIW printing.

**Table 1:** Inks Prepared for DIW 3D Printing from the Combinations of Coll, NFC, CMC, and CA, and Their Final Compositions

inks	NFC (g)	CMC (g)	Coll (g)	CA (g)	the final solid content of each component			
					NFC (g)	CMC (g)	CA (g)	Coll (g)
Coll**0**	50	6	0	10	1.5	6	10	0
Coll**0.1**	50	6	0.1	10	1.5	6	10	0.1
Coll**0.5**	50	6	0.5	10	1.5	6	10	0.5
Coll**1**	50	6	1	10	1.5	6	10	1

### 2.3 DIW 3D Printing

All inks were printed with a BioScaffolder 3.1 (GeSIM, Germany). A 10-mL polyethylene-based plastic syringe (Nordson, U.K Limited) with an inner nozzle diameter of 250 μm was used to dispense the inks to a polystyrene Petri dish (diameter: 5 cm). The syringe was tightly packed with the ink and stored at 8 °C until further use. Circular bioscaffolds (radius 5–7 mm, height: 3, 5, or 8 mm, number of corners at the edge: 100) and cubic bioscaffolds (diameter 25 mm, height 3.5 mm, number of corners at the edge: 4) were printed layer by layer. These dimensions were created with the software GeSIM Robotics BS3.1/3.2. Bioscaffolds were printed by adjusting the dispensing pressure from 140 to 220 kPa and the distance between the strands from 500 to 900 μm. The strand height and width were set to 0.2 mm. The print patterns of each subsequent layer were rotated 90°, and the printing speed was 15 mm/s. For the wet compression tests, bioscaffolds with a radius of 7 mm and a height of 8 mm were printed with the same parameters as those described above. For the dynamic mechanical analysis, bioscaffolds with a cubic shape were used. For micro-CT analysis, SEM, and cell studies, bioscaffolds with a radius of 7 mm and height of 3 mm were printed. For all other analyses, the bioscaffolds had the same size (radius: 7 mm; height: 5 mm).

### 2.4 Freeze-Drying, DHT Treatment, and Neutralization

The printed samples were immediately frozen at −25 °C for 48 h in a freezer and then freeze-dried for 48 h at 10^–3^ mbar and −25 °C. Subsequently, the dry bioscaffolds were cross-linked by DHT treatment at 120 °C for 24 h, as reported elsewhere.^9,27,28^ Then, each cross-linked scaffold was neutralized by immersion in 10 mL of a 0.1 M NaOH solution for 120 min at room temperature. The bioscaffolds were then immersed in 200 mL of ultrapure water (pH 7.4) for 24 h and then rinsed three times with ultrapure water to remove the non-cross-linked CA. The bioscaffolds were then placed on a filter paper and air-dried at room temperature. The cross-linked, neutralized, and air-dried bioscaffolds are referred to as Coll***x***, where ***x*** is the concentration of Coll in the scaffold in wt %.

### 2.5 Characterization Techniques

The morphology of the bioscaffolds (without sputtering) was analyzed by field emission scanning electron microscopy (FE-SEM, Quanta 200 3D, FEI, USA). The images were used to determine pore sizes using ImageJ/FIJI 1.53c software (National Institute of Health, USA).^29^ The morphology of the samples and the micropores in the dry and wet states were analyzed using a SkyScan 1275 X-ray (Bruker, Kontich, Belgium) microcomputed tomography (micro-CT).^30,31^ Attenuated total reflectance Fourier transform infrared (ATR-FTIR) spectra of the bioscaffolds were measured by using a PerkinElmer Spectrum GX Series-73565 FTIR system. Powder X-ray diffraction of the polymers and bioscaffolds was carried out with an X-ray diffractometer (XRD, Bruker D8 Advance, equipped with Cu K_α_ radiation). Thermogravimetric analysis (TGA) was performed using a PerkinElmer TGA 4000 thermal analyzer (Waltham, Massachusetts, USA) in a nitrogen atmosphere. The swelling capacity and weight loss of all bioscaffolds at different time intervals in advanced DMEM at 37 °C were performed, as reported previously.^9,27,30^ Unconfined compression tests were performed under both wet and dry conditions. Samples were measured on a Universal Tester Instron 4204 (Norwood, USA, Instron 2525 Series) and 50 mm compression plates. The dynamic shear moduli of the wet samples were measured on a stress-controlled shear rheometer (Anton Paar MCR 302, Graz, Austria) with a 50-mm parallel plate geometry. Details of all analytical methods can be found in the Supporting Information.

### 2.6 Cytotoxicity Tests

To evaluate how the cells interact with different bioscaffolds, three different in vitro cytotoxicity assays were performed. The cytotoxicity of the sterilized bioscaffolds (with UV–C light) was evaluated using human bone osteosarcoma cells (MG-63; ATCC CRL-1427), which behave similarly to osteoblasts and are commonly used to evaluate biocompatibility and cellular responses to various materials.^32,33^ The MG-63 cells were cultured in Dulbecco’s Modified Eagle’s Medium (DMEM) supplemented with 10% FBS at 37 °C in a humidified atmosphere with 5% CO_2_ and routinely passaged twice per week. For the experiments, the MG-63 cells were seeded in 96-well plates at a seeding density of 7000 cells per well and incubated for 24 h to allow the cells to adhere. They were then treated with bioscaffolds suspended in a fully supplemented cell medium (concentration range: 1–100 μg/mL). After 24 h of treatment, the cytotoxicity of the scaffolds was measured using the Resazurin assay (determination of the metabolic activity of the exposed MG-63 cells), the Coomassie Blue (CB), which measures the amount of cellular proteins proportional to the cell number, and the Neutral Red Uptake (NRU) assay, which measures the lysosomal integrity of the exposed cells. All these assays were performed according to the details published in the literature by Kononenko and Drobne.^34^

For the resazurin assay, 25 μg/mL resazurin was added to each well after cell treatment, and the wells were incubated at 37 °C for 3 h. The fluorescence intensity of the resorufin formed was measured spectrofluorimetrically (BioTek, Cytation 3) at ex/em 560/590 nm. For the NRU assay, 0.04 mg/mL of neutral red dye was added to each well after cell treatment, and the cells were incubated for 2 h to allow the dye to become trapped in the lysosomes of viable cells. The cells were then rinsed with PBS, and the internalized dye was released using a solvent (50% v/v ethanol, 1% v/v acetic acid, and 49% v/v ultrapure water). The released neutral red dye was quantified using a spectrofluorimeter (BioTek, Cytation 3) at ex/em = 530/645 nm. For the CB assay, treated A549 cells were stained with a Coomassie Blue solution (0.05% Coomassie Brilliant Blue G250 in 30% methanol, 10% acetic acid, and 60% ultrapure water) and carefully rinsed with PBS. After rinsing, 0.1 M NaOH was added to the stained cells to dissolve the dye. The optical density of the dissolved CB was measured at a wavelength of 630 nm (BioTek, Cytation 3). Three independent sets of experiments were performed for each cytotoxicity assay with at least four replicates for each treatment condition.

The data from cytotoxicity experiments were expressed as the arithmetic mean ± standard deviation (SD) and were statistically analyzed by the X test (for example: ANOVA with Bonferroni’s posttest for multiple comparisons). A *p* value lower than 0.05 was considered statistically significant. All statistical analyses were performed using Prism 8.4.3 (GraphPad, San Diego, CA, USA).

## 3 Results and Discussion

### 3.1 Ink Preparation, 3D Printing, and Cross-Linking

**Figure 1:**
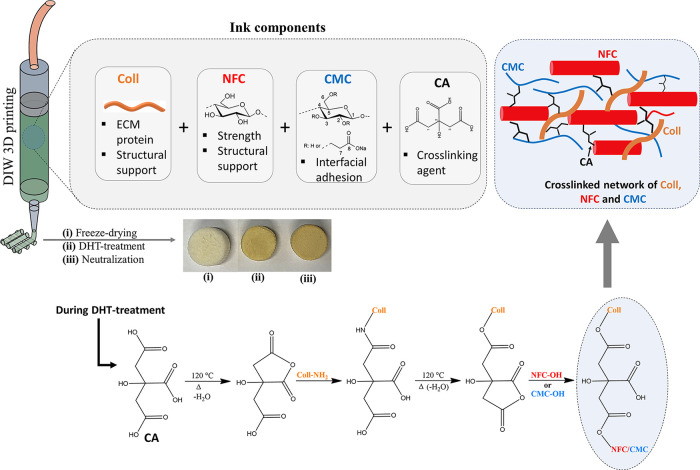
Illustration of ink preparation, DIW printing, scaffolds treatment, and cross-linking mechanism between the components (Coll, NFC, CMC, and CA) in the scaffold.

**Figure 2:**
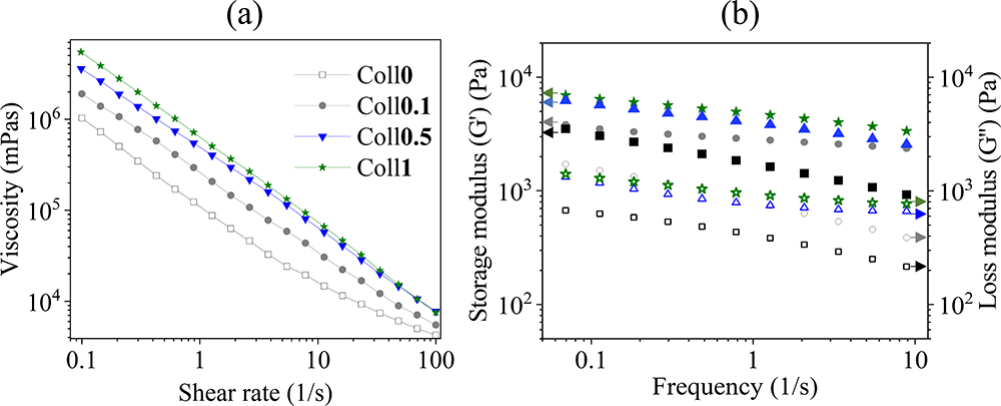
(a) Viscosity and (b) storage and loss modulus of the Coll/NFC/CMC/CA inks prepared with different concentrations of collagen.

The rheological studies are often performed to fine-tune ink formulations for DIW printing in TE applications. The neat collagen (regardless of the concentration) is usually a low viscosity solution and maintaining the right viscosity for printing can be a challenge. Therefore, in this work, hybrid Coll inks (see Figure 1) were prepared from NFC, CMC, CA, and Coll at different concentrations (0.1–1 wt) and investigated for their rheological behavior and suitability for DIW printing. As can be seen in Figure 2a, all investigated inks showed a very strong shear thinning behavior due to the intrinsic interfacial adhesion between NFC/CMC and Coll, which favors the rheological behavior and ensures excellent printability of the inks. In general, the influence of Coll on viscosity is clearly seen and it increased as a function of Coll concentration (0.1–1 wt %). As shown in Figure 2b, all tested inks behave like a rheological gel or a soft solid (the storage modulus *G*′ is higher than the loss modulus *G*′′). Interestingly, the storage modulus and loss modulus are indeed influenced by the Coll concentration and were generally increased by the addition of CA. Optimization and careful formulation are critical to ensure that the ink is suitable for DIW 3D printing and maintains its structural integrity and fidelity during printing. Given the excellent shear thinning properties and higher storage modulus of the coll-based inks presented here, the printability of the presented coll-based inks is comparable to or even better than the coll-based inks described in the literature. These include collagen in combination with the components like ECM,^35^ alginate/fibrin,^36^ gelatin/silk fibroin,^37^ agarose/alginate,^38^ alginate/tannic acid,^39^ polycarprolactone/hydroxyapatite,^40^ and hyaluronic acid.^41^

### 3.2 Scaffold Morphology and Porosity

**Figure 3:**
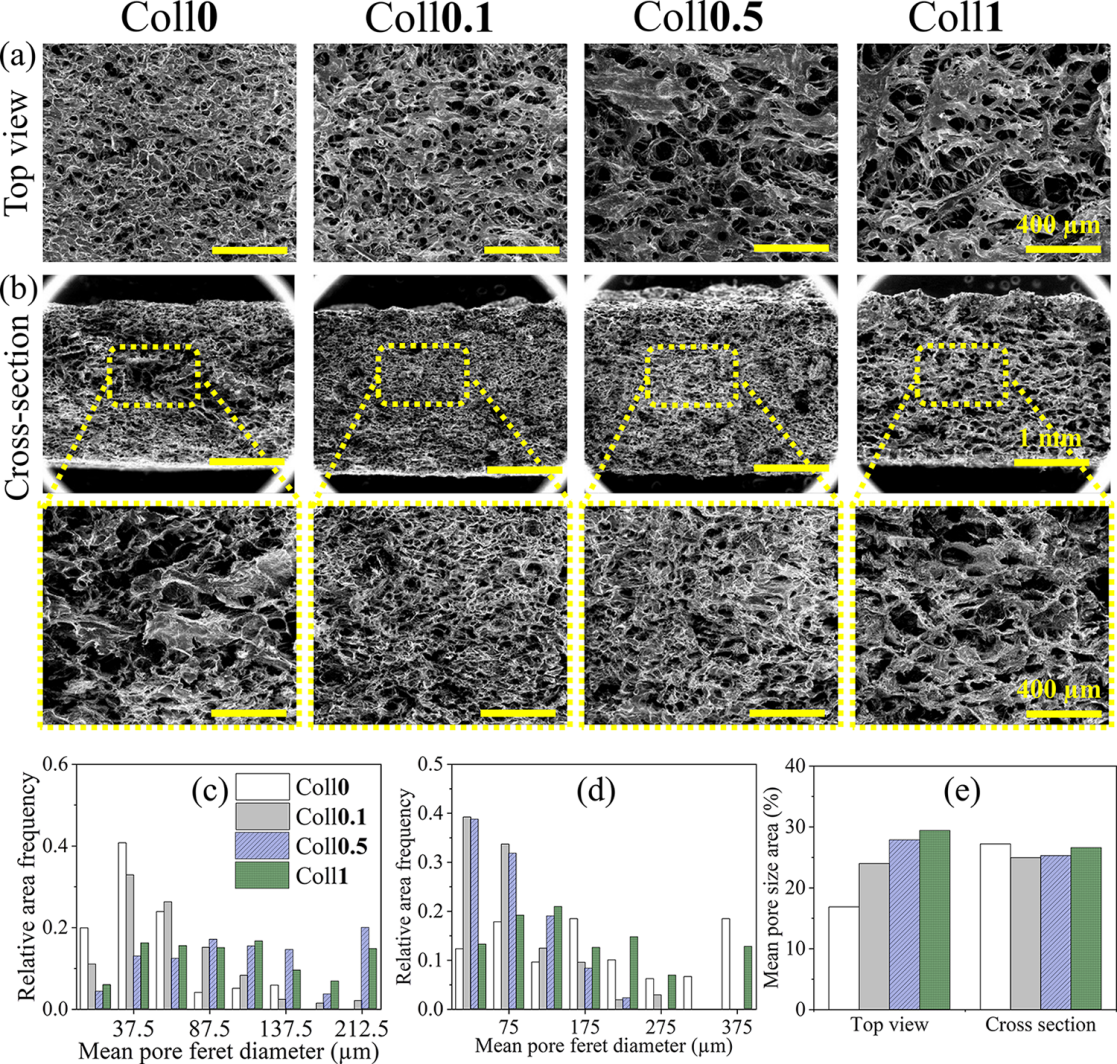
FE-SEM micrographs and pore size analysis. (a) and (b) are the surface (top-view) and cross-section of the DHT treated and neutralized bioscaffolds (Coll*x*, *x* = 0–1 wt %). The mean pore ferret diameter (c: surface, d: cross-section) and pore size area (e) of Coll***x*** scaffold.

The FE-SEM images (top-view and cross-section) of DHT-treated and neutralized (dry) bioscaffolds (Coll***x**, **x*** = 0–1 wt %) are shown in Figure 3. All bioscaffolds showed a porous structure or morphology and interconnected pores on the surface (top-view, Figure 3a) as well as in the cross-section (Figure 3b). The observed pore size ranged from ca. 10 to 220 μm (Figure 3c) on the surface, while in cross-section, it ranged from ca. 25–400 μm (Figure 3d). Although the pore size at the surface of the scaffold increased as a function of Coll concentration, no such behavior was observed in the cross-section (Figure 3e). In general, the observed interconnected pores and variable pore sizes may be beneficial for cell growth and effective nutrient transport, making them an attractive scaffold for TE applications.^13,42,43^

**Figure 4:**
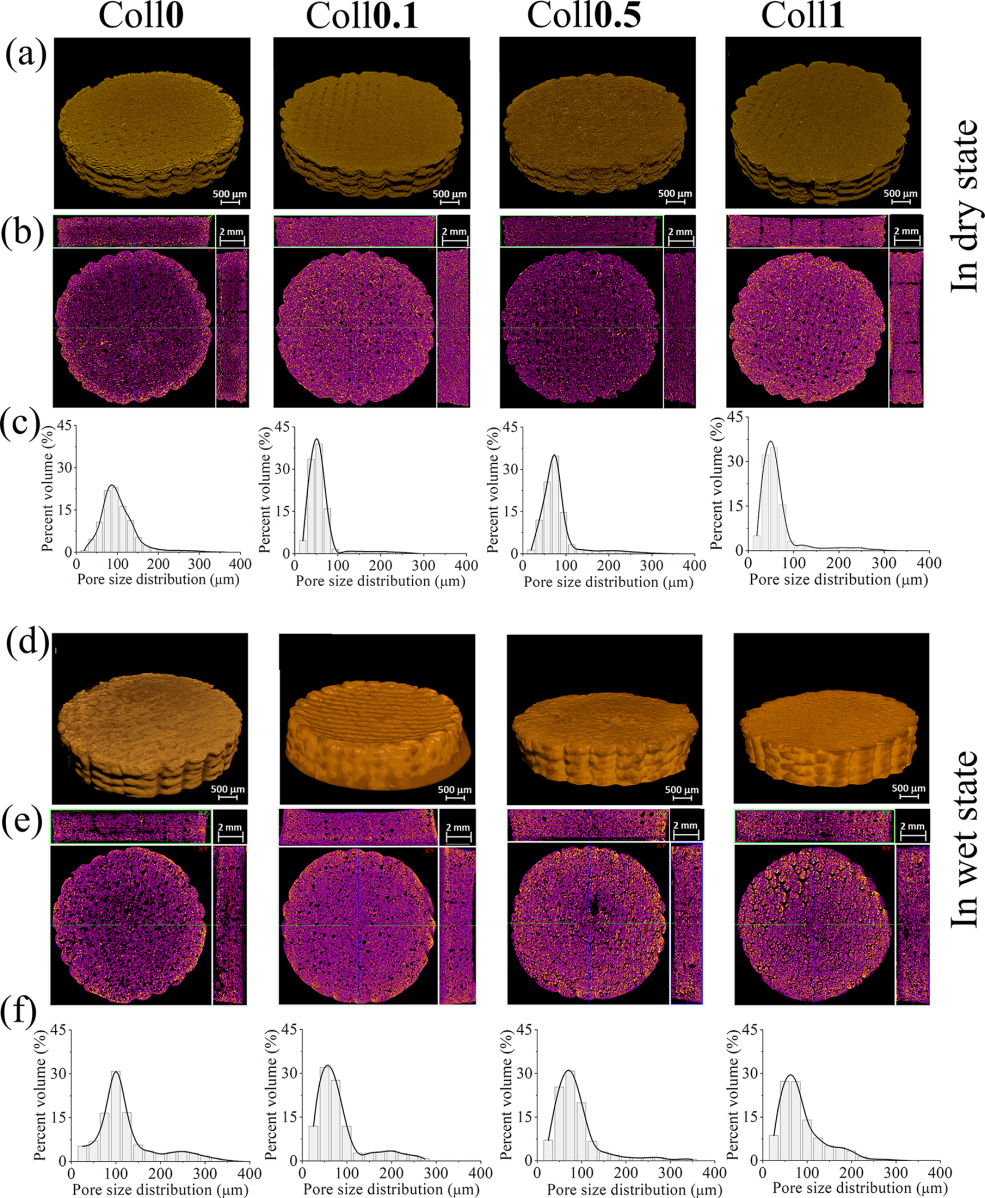
2D (a, d) and 3D micro-CT (b, e) images and pore size distribution profile (c, f) of Coll-free and Coll-containing NFC/CMC/CA bioscaffolds in dry and wet states.

**Figure 5:**
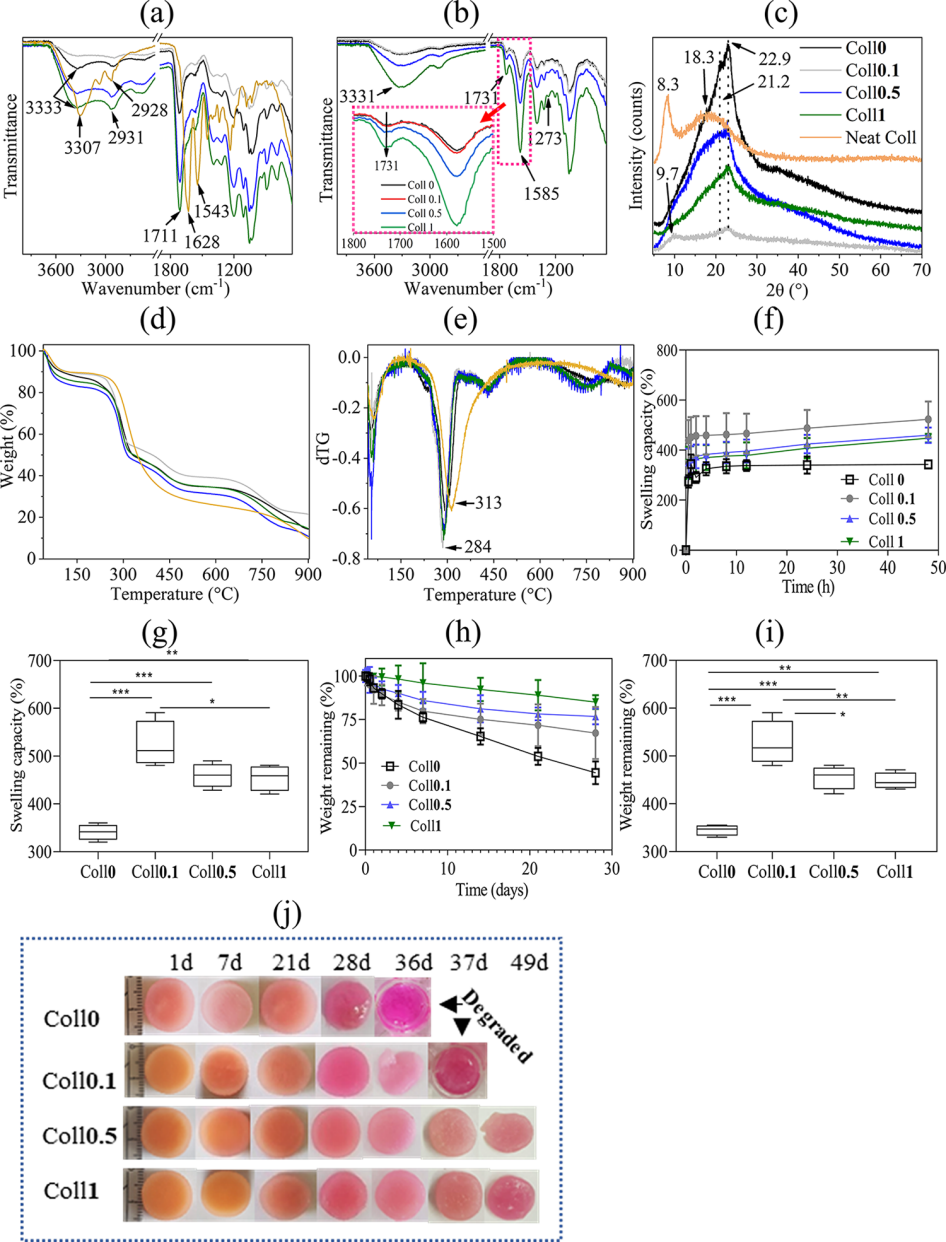
(a, b) ATR-FTIR spectra, (c) XRD diffractograms, (d, e) TGA and dTG curves of neat and Coll bioscaffolds (Coll*x*, ***x*** = 0–1 wt %) before and after DHT treatment and neutralization. Swelling (f, g) and degradation (h, i) of the DHT-treated and neutralized bioscaffolds (Coll***x**,**x*** = 0–1 wt %) in biofluid at 37 °C. (j) Images of Coll-free and Coll scaffold after being immersed in biofluid at 37 °C at different time periods. Data analysis was done by one-way ANOVA with Tukey’s multiple comparison test. Values are presented as mean ± standard deviation (SD); *p < 0.05, **p < 0.01, ***p < 0.001 (compared to control Coll**0**).

Micro-CT measurements for all bioscaffolds in dry and wet conditions were performed to analyze morphology, pore size, porosity (open and closed), and wall thickness. The 3D and 2D reconstruction image analysis of the dry bioscaffolds (Coll***x**,**x*** = 0–1 wt %, (Figure 4) clearly shows the pore distribution in 2D (Figure 4a) and the interconnectivity of the porous structure. The mean pore diameter and mean wall thickness of the Coll-free scaffold (Coll**0**) were about 102.8 μm, and the total porosity (or open porosity) was about 78.8% (Table 1). The latter is defined as the volume fraction of the interconnected void space within a scaffold. The addition of Coll resulted in a decrease in the structural parameters. For example, the mean pore size and total porosity decreased from ca. 102.8 μm (Coll**0**) to 59.5–80.7 μm (Coll**0.1**-**1**) and from 78.8% (Coll**0**) to 53.1–66.5% (Coll**0.1**-**1**), respectively. This reduction in the mean pore size or total porosity could be because the gaps between the scaffold pores in Coll**0** were filled by Coll fibers, which reduces their size.^4^ Such a reduction in the pore size can lead to a denser and structurally more stable scaffold, as observed in the weight loss test (see Figure 5i, j). This is in line with the results published by other authors for Coll-based scaffolds.^44,45^ Among the Coll-containing bioscaffolds, Coll**0.5** showed an increased total porosity/pore size and wall thickness. The observed values for closed porosity were in the range of 0.00–0.01%. These very low values indicated the presence of highly interconnected pores within the scaffold.^28,46^ In general, closed porosity refers to the volume fraction of the isolated void space within a scaffold, which is not connected to the open pores.^31^ The pore size distribution profile (Figure 4c) shows that the pore sizes were in the range of 20–80 μm (22%), 100–200 μm (5–15%), and 200–350 μm (<0.6%) for Coll**0**. Interestingly, for all Coll bioscaffolds, the sizes of the smaller pores (20–80 μm) were about 15% higher, and the larger pores (100–350 μm) were about less than 1% compared to the Coll-free scaffold. The overall pore size and porosity in the range of 20–100 μm and 53–79% are suitable for skin TE or neovascularisation, as suggested by other authors.^47,48^ In general, the pore size values obtained with the micro-CT were lower than those obtained with the FE-SEM.^28^ A possible reason for this could be that the assessment of 3D pore size in micro-CT is based on a sphere fitting algorithm. This is the most accurate parameter considering the whole specimen evaluation, orientation-dependent direct 3D analysis, low image processing bias, irregular pore assessment, and lack of subjectivity in the assessment.^31,49^ On the other hand, the FE-SEM analysis is limited to a 2D structure and a certain number of sections due to the mechanical sectioning and the special treatment of the sample.^28,50^ Furthermore, the lateral resolution of the micro-CT is lower than that of the FE-SEM, which limits the detection of fine pore intersections.^51^ These variables could cause structural changes and make it difficult to accurately determine the pore margin and connectivity, and thus, the pore size/porosity determined using micro-CT is usually lower than the results obtained using FE-SEM.^28,49^

The results of the micro-CT measurements carried out in the wet state are shown in Figure 4d–f and Table 1. This was done to determine the structural changes associated with hydration. For this purpose, all bioscaffolds were immersed in water (pH 7.4) at 37 °C for 24 h prior to the experiment, and then the experiments were also conducted in water for about 3 h. A significant change in morphology, i.e., pore size/porosity, was observed in the hydrated bioscaffolds compared to the dry bioscaffolds (see Figure 4a, b). For example, the total porosity of the hydrated bioscaffolds decreased significantly from 53 to 79 to 2.7–5.6%, while the pore size and pore wall thickness increased more than three times. One explanation could be that the Coll fibers swollen by hydration occupy more space within the bioscaffolds, resulting in larger pore size and a decrease in overall porosity.^46^ During hydration, the Coll fibers may reorganize, possibly leading to a change in pore structure size, contributing to an increase in pore size but a decrease in overall porosity.^46^ Although the total porosity increased with increasing Coll concentration in the scaffold, no major differences in pore size and pore wall thickness were observed. It was reported previously that an increase in pore size in the wet state can be beneficial for cell growth and tissue development.^46^ The results of the pore size distribution profiles (Figure 4f) showed that the hydrated scaffold is close to cartilage (e.g., ear) and bone (e.g., cortical) tissue regeneration in terms of pore size (25–100 μm, 11–27%, and 100–350 μm: 3–11%).^43^ Thus, by means of structural parameters in the hydrated state, Coll bioscaffolds have the potential to be used as biotemplates and bioscaffolds for TE applications.

### 3.3 Composition, Structure, Charges, Thermal, Degradation, and Swelling Properties

The ATR-FTIR spectra of the neat Coll and Coll bioscaffolds (Coll*x*, ***x*** = 0–1 wt %), before and after DHT treatment and neutralization, are shown in Figure 5a and 5b (see also Table S1). The neat Coll (before and after DHT treatment) showed characteristic peaks at 3303 (amide A: Coll I and II), 2927 (amide B: Coll I and II), 1630 (amide I: C=O stretching vibrations), 1544 (amide II: C–N stretching vibration), 1238 (amide III: N–H bending, C–N stretching and N–H in-plane bending vibration), and 3333 cm^–1^ (OH stretching vibrations).^52^ In the case of all bioscaffolds and before DHT treatment, the characteristic peaks for polysaccharides (NFC and CMC) were observed at 3347 (OH stretching vibrations), 2898 (C–H stretching vibrations), 1394 (COO^–^ stretching vibrations), 1326 (C–O stretching vibrations) and 1055 cm^–1^ (C–O–C stretching).^53^ The two peaks observed at 1711 and 1585 cm^–1^ can be related to the carbonyl (C=O) vibrations of CA and CMC. The presence of Coll in the bioscaffolds can be confirmed by the amide I and amide II peaks at 1630 (C=O stretching vibrations) and 1544 (amide II: C–N stretching vibration) cm^–152^. All these peaks were also detected for DHT-treated and neutralized bioscaffolds. In addition, the amide bonds (I, II, and III) at 1630, 1544, and 1238 cm^–1^ of Coll were also detected, confirming that Coll is still present in the bioscaffolds (see Table S1). A new peak at 1730 cm^–1^ corresponding to ester carbonyl was also detected, which is due to the cross-linking of carboxyl groups of CA with the hydroxyl groups of NFC, CMC, or Coll.^28^ This ester peak is more pronounced with a higher concentration of Coll in the scaffold (Figure 5b, inset), while the peaks of all other components (NFC, CMC, and CA) remained unchanged (see Table S1). One explanation could be that the higher concentration of Coll favored the formation of more ester bonds between CA and Coll.

Figure 5c shows the XRD diffractograms of neat Coll and Coll bioscaffolds (Coll*x*, ***x*** = 0–1 wt %, DHT treated and neutralized). The neat Coll shows the characteristic diffraction peaks at 2θ = 8.3 and 20.9, which are characteristic of disordered Coll fibrils.^54^ For NFC, four main diffraction peaks were found at 2θ = 14.6°(110), 20.1°(020), 34.2°(004) and a diffraction pattern corresponding to crystalline cellulose I.^9,54^ CMC, on the other hand, showed a broad diffraction peak at 2θ = 20° and an amorphous structure (see Figure S1).^55^ Interestingly, although the typical diffraction peaks of NFC were found, the main peak of Coll and CMC at 2θ = 20–21° was covered by the diffraction peak of NFC in all bioscaffolds and no new peaks were present, indicating that the structural properties of the bulk phase of all components were preserved after DHT treatment.

Figure 5d, e shows the results of the TGA and its derivative (dTG, mass loss rate) of the neat Coll and the bioscaffolds with different Coll concentrations between 40 and 900 °C. It can be seen that the thermogram (or degradation pattern) of the neat Coll differs from that of the Coll bioscaffolds. However, no significant differences in degradation pathways were observed between the different Coll concentrations in the scaffold. There were two main degradation steps for both the neat Coll and the Coll-containing bioscaffolds. The first degradation stage occurred between 40 and 110 °C, and the curve showed a peak in dTG at 64 °C and between 54 and 56 °C. This can be attributed to the removal of physically absorbed water.^9,53^ The latter corresponds to 9–15% of the sample weight. In the second degradation phase, i.e. from 190 to 500 °C, a peak in dTG was observed at 313 °C for neat Coll and between 285 and 296 °C for Coll bioscaffolds. The observed peaks in dTG for increasing amounts of Coll in the bioscaffolds were in the following order: Coll**0** > Coll**0.5** > Coll**1** > Coll**0.1** (Figure 5e). This suggests that the dTG peak was slightly shifted to a lower temperature (284 °C) by the incorporation of Coll, which could be due to the degradation and denaturation of Coll. The observed weight loss in the second step was 58, 61, and 56–61% for neat Coll, Coll-free bioscaffolds, and bioscaffolds with different amounts of Coll, respectively. These values were lower than the mass loss of CA (95%) and NFC (82%) and similar to those of neat CMC (58%, Figure S2) and neat Coll (69%). This shows that the different Coll concentrations in the bioscaffolds do not significantly impact the thermal stability of the bioscaffolds, despite large differences in the mechanical properties (see Section 3.4).

The swelling capacity is an important indicator of the suitability of bioscaffolds for TE applications, as it can provide the necessary aqueous environment and facilitate the transfer of cell nutrients and metabolites, etc.^56,57^ The swelling behavior of all cross-linked and neutralized bioscaffolds (Coll*x*, ***x*** = 0–1 wt %, Figure 5f,g) was investigated in the cell growth medium (biofluid) at 37 °C. Figure 5f shows that the uptake of all bioscaffolds into the biofluid increased rapidly in the first hour (0–1 h) and slowed down in the following hours. For the Coll-free scaffold (Coll**0**), a steady state was reached after 8 h compared to the other bioscaffolds. All Coll-containing bioscaffolds (0.1–1.0 wt %) still did not show a steady state after 48 h. Interestingly, all Coll bioscaffolds showed a significantly higher swelling capacity compared with the Coll-free scaffold (Coll**0**, Figure 5g). This could be due to the presence of various hydrophilic functional groups (hydroxyl, carboxyl, amine) in Coll, which can bind more water molecules and thus increase the swelling capacity.^58^ Within the Coll bioscaffolds, the bioscaffolds containing a higher Coll concentration (Coll**0.5** and Coll**0.1**) showed slightly less swelling. This could be due to the formation of a tighter network structure, the consumption of the hydrophilic functional groups of Coll, and the reduced pore size (Table 2) in the wet state, which resulted in less diffusion of the biofluid and thus less swelling. Overall, the swelling capacity of the Coll bioscaffolds increased to 53% compared to Coll-free bioscaffolds. Regarding the swelling capacity of the bioscaffolds, the following order was found (Figure 5g): Coll**0.1** (523 ± 71 (g/g)) > Coll**0.5** (460 ± 30 (g/g)) > Coll**1** (447 ± 16 (g/g)) > Coll**0** (343 ± 15 (g/g)).

**Table 2:** Summarizes the Main Structural Parameters (Total/Open and Closed Porosity Volume in %, Average Pore Size, Wall Thickness) from the 3D Analysis of the Coll-Free and Coll-Containing Bioscaffolds

samples	total/open porosity (%)	closed porosity (%)	pore size (μm)	wall thickness (μm)
in dry condition				
Coll**0**	78.82	0.00	102.81	45.93
Coll**0.1**	54.98	0.01	59.52	47.95
Coll**0.5**	65.50	0.00	80.67	49.20
Coll**1**	53.10	0.01	65.67	52.77
in wet condition				
Coll**0**	5.42	6.87	121.60	327.37
Coll**0.1**	2.71	5.49	83.02	226.62
Coll**0.5**	4.13	7.73	87.78	233.70
Coll**1**	5.56	6.89	87.98	219.00

The results of in vitro degradation of bioscaffolds (Coll*x*, ***x*** = 0–1 wt) in the presence of biofluid at 37 °C and at different time periods are shown in Figure 5h,i. Not all bioscaffolds were completely degraded after 28 days (Figure 4j). The Coll-free scaffold was more susceptible to degradation and showed a mass loss of 56% after 28 days (Figure 5). The Coll**1** scaffold showed the highest stability, with a mass loss of only 15% after 28 days. The observed mass losses for Coll**0.1** and Coll**0.5** were 33 and 23%, respectively. All bioscaffolds showed a gradual decrease in mass over time. Interestingly, the bioscaffolds’ degradation rate decreased with increasing Coll concentration. It is assumed that the increased cross-linking density of Coll with CA improves the stability of the scaffold and thus reduces the mass loss. However, when it comes to in vivo experiments, the stability achieved here through in vitro experiments may hinder the formation of new tissue. In addition, the stability of hybrid bioscaffolds could be reduced under in vivo conditions where the scaffolds come into contact with the selected tissue type, multiple enzymes, microenvironments, etc. This could limit the use of hybrid bioscaffolds for long-term in vivo TE applications. To verify this and find a balance between scaffold stability and tissue regeneration, in vivo degradation studies are required and will be performed as part of future work.

### 3.4 Mechanical Properties

**Figure 6:**
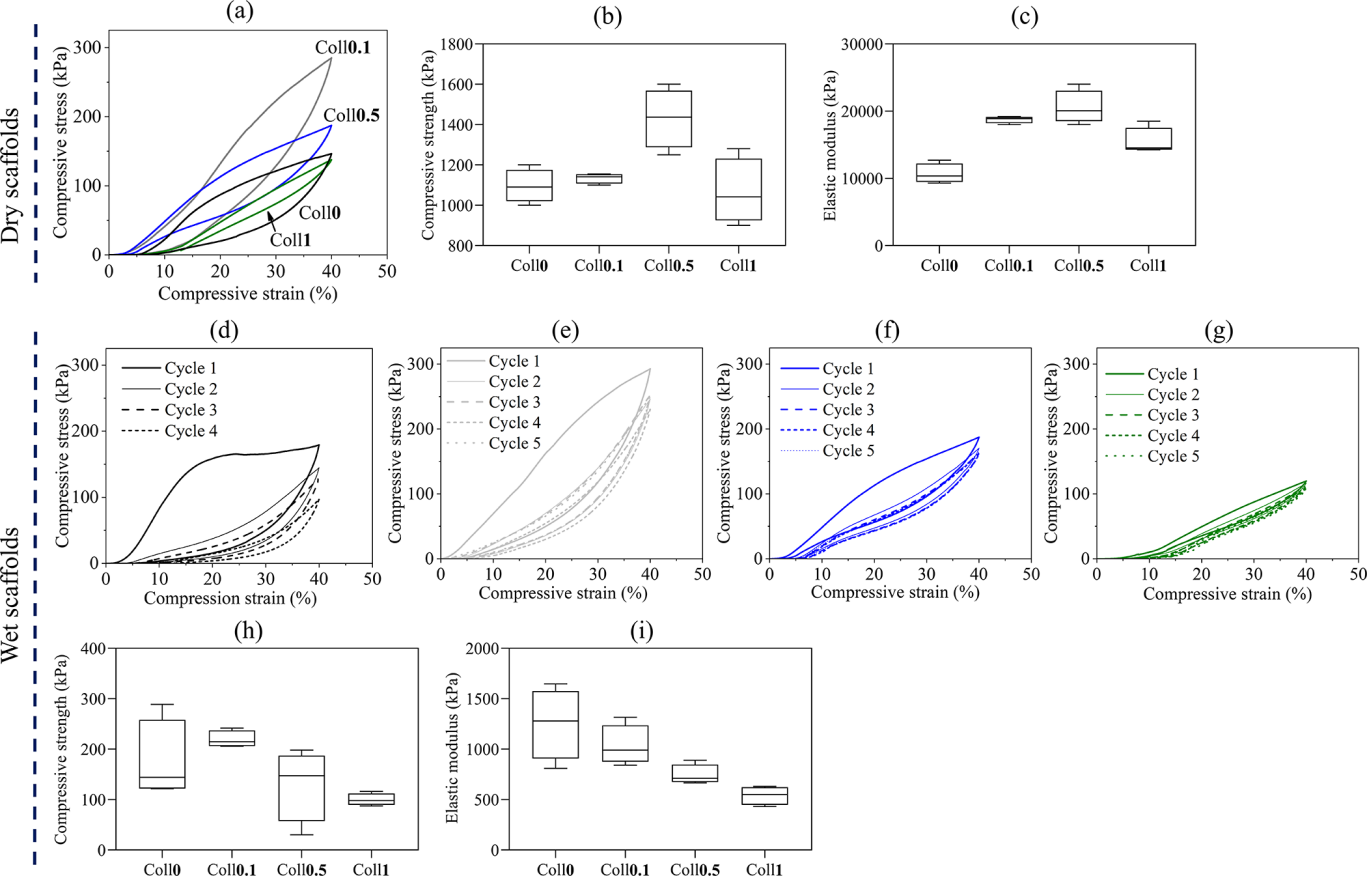
Compressive mechanical properties of dry and wet cross-linked bioscaffolds (with and without Coll). Compressive stress vs strain curves (a), compressive strength (b: at 30% strain), and elastic modulus (c) of dry bioscaffolds. Cyclic compressive curves of Coll**0** (d), Coll**0.1** (e), Coll**0.5** (f), Coll**1** (g), and compressive strength (h: at 40% strain) and elastic modulus (i) of wet bioscaffolds.

The mechanical properties of all bioscaffolds were investigated by using unconfined compression tests in both dry and wet conditions. Figure 6a–c shows the mechanical compression properties of the dry bioscaffolds without Coll (Coll-free) and the bioscaffolds with Coll (Coll-composite). The addition of Coll resulted in improved mechanical performance compared to the bioscaffolds without Coll. As shown in Figure 6a, the Coll**0.5** scaffold had the highest compressive strength (measured at 30% strain) of 1473 and 1871 kPa. The compressive strength of all the bioscaffolds ranged from 1.0 to 1.9 MPa. Interestingly, the compressive strength increased slightly with increasing Coll concentration (Coll**0.1** and Coll**0.5**), followed by a decrease for Coll**1**. This trend was also reflected in the elastic modulus, which ranged from 10 to 20 MPa. Remarkably, our dry and cross-linked Coll bioscaffolds had comparable or better elastic moduli than other Coll composite bioscaffolds reported in the literature (Coll/Chitosan: 377–524^59^ and 122–563 kPa,^60^ Coll: 15 kPa,^61^ Coll/Hyaluronic acid: 35–95 kPa,^62^ Coll/Polylactide/Bioapatite/hyaluronic acid: 2–21 MPa.^46^

The wet compressive mechanical properties of the Coll bioscaffolds are illustrated in Figure 6d–i. In general, the mechanical properties of Coll bioscaffolds were lower than those of Coll-free bioscaffolds and decreased with higher Coll concentrations. The average elastic modulus decreased by 2.4 times from 1272 kPa (Coll**0**) to 521 kPa (Coll**1**), while the average compressive strength increased from Coll**0** to Coll**0.1** and decreased with higher Coll content. Coll increased the reproducibility of the sample preparation (lower standard deviation); as the elastic modulus is decreased, the samples become more flexible, which is also reflected in the shape of the compression curve and its deviation in repeated compression tests (Figure 6d–g). The latter figures show relaxation curves depicting all samples recovering their dimensions effectively under compression up to 40% strain. Furthermore, the profile of the compression curve changed after the initial cycle, and the elastic response continuously decreased for all bioscaffolds. Interestingly, increasing Coll concentration in the bioscaffolds correlated with improved elastic response—lower hysteresis and dissipated energy compared to Coll**0**. For example, the difference in compression strength between the first and last cycle was 82 kPa for the Coll**0** scaffold, while it was only 12 kPa for Coll1. Considering the highest compression strength was attained at Coll**0.1**, these results suggest that at low Coll content, Coll maintains cohesion between NFC while increasing flexibility of the sample. This is also related to the availability and spatial distribution of NFC, CMC, and Coll cross-linked with CA. A higher amount of Coll seems to result in lower NFC/matrix interactions. The compressive strength of our hydrated Coll scaffolds remains on par with or surpasses what was reported for related scaffolds such as Coll/Silk fibroin (14.7 kPa).^63^ Our Coll scaffold’s elastic modulus also surpasses that of other Coll bioscaffolds, such as Coll/silk fibroin (35–50 kPa),^63^ Coll/chitosan ((100 kPa),^64^ (6–18 kPa),^60^ Coll (1.55–42 kPa^65^). Notably, human articular cartilage presents similar and higher elastic modulus values, ranging from 0.02 to 1 MPa.^66−69^

### 3.5 Cytotoxicity

**Figure 7:**
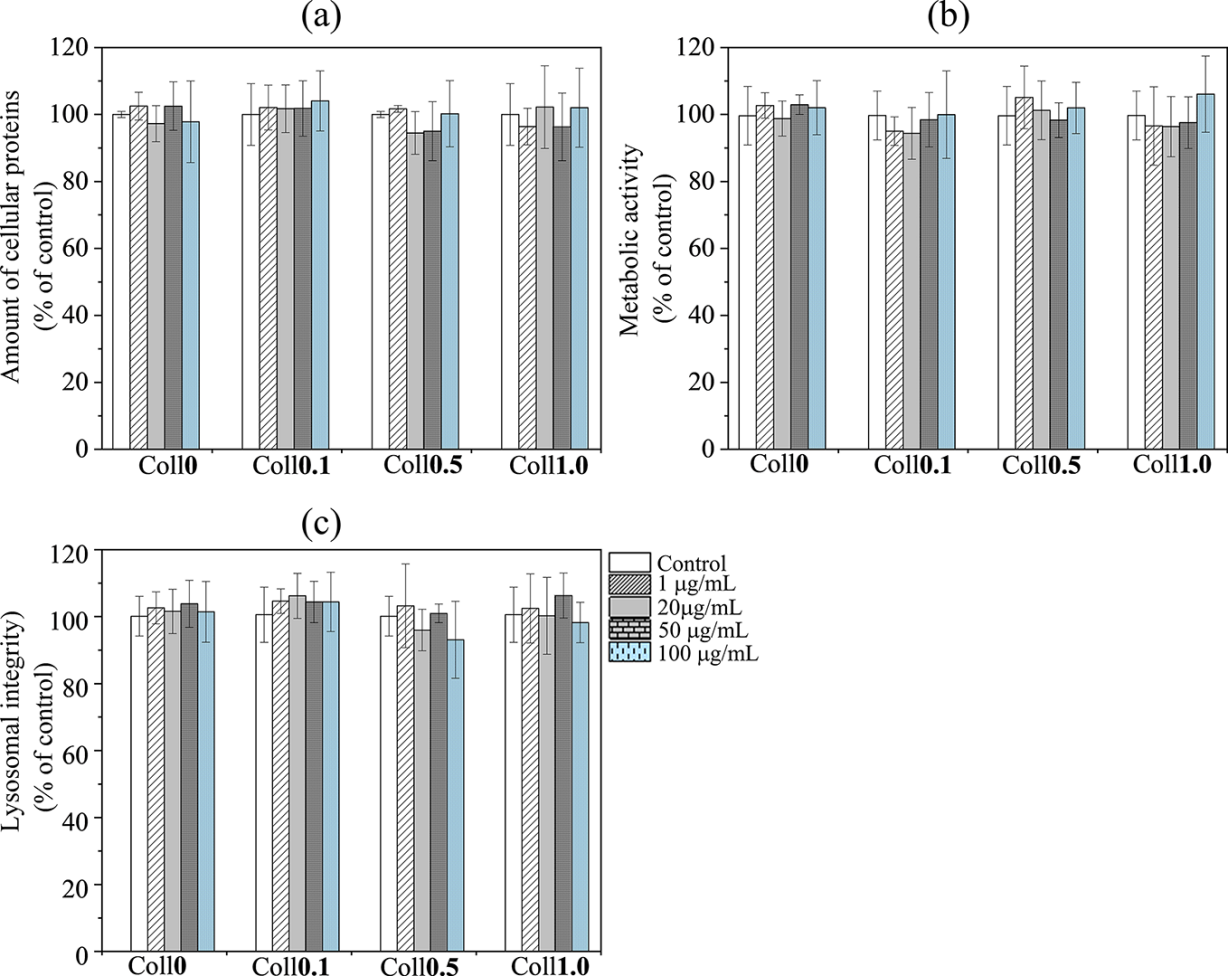
Cytotoxicity of bioscaffolds exposed to MG-63 human osteosarcoma cells at different concentrations. Assessment of different cytotoxicity assays (a: Coomassie Blue, b: Resazurin assay, c: Neutral red uptake) was performed after 24 h of exposure to bioscaffolds.

In vitro cell culture tests were performed to evaluate the potential hazards of our bioscaffolds with human bone osteosarcoma cells (MG-63). The safety was estimated using three in vitro cytotoxicity assays (resazurin assay, Coomassie blue assay, and NRU assay) to determine whether the different bioscaffolds at different concentrations (1–100 μg/mL) had adverse effects on the MG-63 cells.^70^ The CB assay showed that none of the bioscaffolds significantly affected the cell number (Figure 7a). The resazurin assay showed that the treatments used in this study did not affect the metabolic activity of the exposed cells (Figure 7b). As shown by the NRU assay (Figure 7c), the scaffold had no significant effect on the lysosomal integrity, indicating low hazard. Overall, the results of all tests showed no cytotoxicity of the Coll-free or Coll-containing bioscaffolds, during the 24 h experimental period. This suggests that the cross-linked and porous Coll-NFC-CMC composite bioscaffolds are good candidates for use in TE. It is vital to note that our study’s primary aim was to evaluate cytotoxicity rather than cell proliferation. Scaffold optimization and extensive cell testing are further required for a comprehensive and long-term in vitro cell growth analysis (proliferation) or in vivo experiments. This will be performed in the future work.

## 4 Conclusions

This work reports on the preparation and characterization of chemically cross-linked Coll-nanocellulose hybrid bioscaffolds. An ink containing Coll in different concentrations, NFC, CMC, and CA, was prepared and 3D printed. The printed bioscaffolds were freeze-dried and then DHT was treated and neutralized. The quality of the freshly printed strands was examined with an optical microscope. CA, a green and inexpensive cross-linker, was used to cross-link Coll, NFC, and CMC in the bioscaffolds via ester bonds at elevated temperatures and in the dry state. SEM measurements showed that the cross-linked and dry bioscaffolds had pore sizes ranging from about 10 to 400 μm, which is lower than the pore sizes determined by micro-CT (65–102 μm). The observed pore size/porosity for the Coll bioscaffolds was higher in the wet state, as determined by micro-CT. In general, all Coll bioscaffolds had lower pore size or porosity compared to Coll-free bioscaffolds, as shown by the SEM and micro-CT analyses. The formation of ester bonds or cross-link density of CA with Coll/NFC/CMC was increased as a function of Coll, as shown by ATR-FTIR. It was found that the swelling and degradation properties of Coll-containing bioscaffolds were increased compared to Coll-free bioscaffolds. These properties were further controlled by tailoring the amount of Coll within the structure. A lower Coll amount (Coll0.1) exhibited higher swelling and lower degradation compared to the other two amounts (Coll0.5 and Coll1). The higher the Coll amount in the scaffold, the greater the dimensional stability (with no complete collapse). Interestingly, no major changes in thermal and structural properties were observed as a function of the Coll amount, as demonstrated by TGA and XRD analyses. The mechanical compressive properties of the cross-linked Coll bioscaffolds increased compared to the Coll-free bioscaffolds but decreased with increasing Coll concentration. This behavior was observed for both the dry and wet bioscaffolds. All bioscaffolds showed exceptional dimensional stability when exposed to a complex biological fluid (cell growth medium), and the bioscaffolds with higher Coll(1%) concentrations were stable for up to 48 days. The interaction of human bone osteosarcoma cells with Coll hybrid bioscaffolds (tested with different assays) showed that the bioscaffolds were noncytotoxic. However, further in vivo studies are required to validate the potential applicability of our hybrid bioscaffolds in TE applications. This is a crucial step in translating our work into the field of cartilage TE, where chemically cross-linked collagen-based hybrid scaffolds are required to ensure load-bearing capacity and tissue repair capability.

## Supporting Information Available

Detailed all analytical methods (SEM, micro-CT, ATR-IR, powder XRD, TGA, swelling and weight loss, compressive mechanical strength testing, peaks from IR results and results from the XRD and TGA of neat polymers (PDF)
