# Supplementary material for: 3D-Printed Collagen–Nanocellulose Hybrid Bioscaffolds with Tailored Properties for Tissue Engineering Applications
Source: ACS Appl Bio Mater. 2023 Dec 5;6(12):5596–608. doi: 10.1021/acsabm.3c00767 (PMC10731651; doi:10.1021/acsabm.3c00767)
Supplement: Supplementary file 1 — mt3c00767_si_001.pdf [file mt3c00767_si_001.pdf]

# Supporting Information

# 3D printed collagen-nanocellulose hybrid bioscaffolds with tailored properties for tissue engineering applications

Andreja Dobaj Štiglic[1,8], Florian Lackner[2], Chandran Nagaraj[3], Marco Beaumont[4], Matej Bračič[1], Isabel Duarte[5], Veno Kononenko[6], Damjana Drobne[6], Balaraman Madhan[7], Matjaž Finšgar[8], Rupert Kargl[1,2], Karin Stana Kleinschek[2,9,*], Tamilselvan Mohan[1,2,*]

[1]University of Maribor, Faculty of Mechanical Engineering, Laboratory for Characterization and Processing of Polymers, Smetanova ulica 17, 2000 Maribor, Slovenia

[2]Graz University of Technology, Institute of Chemistry and Technology of Biobased System (IBioSys), Stremayrgasse 9, 8010 Graz, Austria

[3]Ludwig Boltzmann Institute for Lung Vascular Research, Stiftingtalstrasse 24, Graz, 8010 Austria

[4]Department of Chemistry, Institute of Chemistry o Renewable Resources, University of Natural Resources and Life Sciences Vienna (BOKU), A-3430 Tulln, Austria

[5]Department of Mechanical Engineering, Centre for Mechanical Technology and Automation (TEMA), Intelligent Systems Associate Laboratory (LASI), University of Aveiro, 3810-193 Aveiro, Portugal

[6]Biotechnical Faculty, Department of Biology, Večna pot 111, 1000 Ljubljana, Slovenia

[7]CSIR-Central Leather Research Institute, Chennai 600 020, Tamil Nadu, India

[8]University of Maribor, Faculty of Chemistry and Chemical Engineering, Laboratory for Analytical Chemistry and Industrial Analysis, Smetanova ulica 17, 2000 Maribor, Slovenia

[9]University of Maribor, Institute of Automation, Faculty of Electrical Engineering and Computer Science, Koroska cesta 46, 2000 Maribor, Slovenia

**Correspondence*: tamilselvan.mohan@tugraz.at, karin.stanakleinschek@tugraz.at

## Analytical methods

### 1.1 Rheology

The rheological properties of all inks were determined with a Modular Compact Rheometer (MCR 302, Anton Paar, Germany) at 25° C. Viscosity curves were measured from a shear rate of 1 to 100 $s^{-1}$ . Frequency sweeps were evaluated at constant strain of 0.1% (in the linear viscoelastic region) in frequency window of 0.1 to 10 $s^{-1}$ .

### 1.2 Field emission scanning electron microscopy (FESEM)

Prior to SEM imaging, all samples were pressed onto a double-sided carbon adhesive tape (SPI 116 Supplies, USA). No sputtering was performed on the sample surfaces. The freeze-dried scaffolds were immersed into liquid nitrogen and fractured to analyze the cross-section of the samples. The images were recorded with an acceleration voltage of 10 kV in low vacuum conditions (60 Pa) at room temperature. For the image analysis of the SEM images the software ImageJ/FIJI 1.53c (National Institute of Health, USA) [1] was used by generating a binary image and subsequently using the inbuild particle measurements. Pores smaller than 10 $pixel^2$ (approx. 63 $\mu m^2$) were excluded from the calculation to avoid miscounts from single pixels and artifacts.

### 1.3 Microcomputed tomography (µCT)

Samples were analyzed in a X-ray microcomputed tomography (µCT) equipment from SkyScan 1275 (Bruker µCT, Kontich, Belgium) with penetrative X-rays of 36 kV and 235 µA, in high resolution mode with a pixel size of 9.5 µm, 58 ms of exposure time, 5 of frame averaging, 0.20

deg of rotation step and 360° of rotation. NRecon (v.1.7.3.1 software, Bruker, Kontich, Belgium) and CTVox (v.3.3.0 r1403 software, Bruker, Kontich, Belgium) softwares were used for 3D-reconstruction and CTAn software (v.1.17.7.2 software, Bruker, Kontich, Belgium) was used in morphometric analysis (e.g., porosity values and pore size distribution), in which the images were segmented and analyzed. Herein, the thresholding or image binarization/segmentation was performed using a global thresholding technique for each sample which employs a fixed range of greyscales (lower and upper scales set at 69 and 255) for both foreground (white) and the pixels outside of the range area, which are set as the background (black).

**Wet (µCT) measurements**

Samples were immersed in MilliQ-water for 24 h, respectively. Afterwards, the sample were analyzed with penetrative X-rays of 50 kV and 200 µA, in high resolution mode with a pixel size of 12.5 µm, 30 ms of exposure time, 5 of frame averaging, 0.20 deg of rotation step and 360° of rotation.

## 1.4 Attenuated Total Reflection-Fourier Transform Infrared (ATR-FTIR) Spectroscopy

The ATR-FTIR spectra of scaffolds were measured using a Perkin Elmer FTIR System Spectrum GX Series-73565 at a wavenumber range of 4000 – 400 cm$^{-1}$. A total of 32 scans were performed for all measurements with a resolution of 4 cm$^{-1}$.

## 1.5 Powder X-Ray Diffraction (XRD)

The powder X-ray diffraction of polymers and scaffolds was investigated with an X-ray diffractometer (XRD, Bruker D8 Advance equipped with Cu Kα radiation). The scaffolds were cut into small pieces and deposited on the sample holder, and the XRD patterns were recorded

at room temperature between a scattering angle (2θ) 4° to 70° and steps of 0.02°, and a scan rate of 0.02° 2θ $s^{-1}$.

### 1.6 ThermoGravimetric Analysis (TGA)

The TGA was performed on a TGA 4000 thermal analyzer from Perkin Elmer (Waltham, Massachusetts, USA) instrument in a nitrogen atmosphere (20 mL $min^{-1}$) of 40 to 900 °C at a heating rate of 10 °C $min^{-1}$ using an $Al_2O_3$ crucible without a lid. The Pyris software, Version 10.02.0468, was used for data evaluation.

### 1.7 Analysis of swelling capacity and weight loss

The swelling kinetics of the neutralized scaffolds in cell culture medium were investigated using a gravimetric method. The dried cylinder-shaped scaffolds ($d$ = 14 mm, $h$ = 5 mm) were weighed (initial weight, $W_0$), immersed in 5 mL biofluid (pH 7.4) at 37 °C. At predetermined time intervals ($W_t$), the scaffolds were removed from the liquid, wiped dry carefully by a filter paper only on the surface and weighed again. The swelling capacity at time $t$ was calculated using Equation (1).

$$\text{Swelling capacity (\%)} = \frac{\text{Wt}-\text{Wo}}{\text{Wo}} \text{x } 100 \qquad (1)$$

To determine the weight loss upon contact with bioliquid, the scaffolds (initial weight, $W_0$) were placed in a beaker with 5 ml of biofluid at 37 °C and stirred at 200 rpm. At predetermined intervals, the scaffolds were removed from the biofluid, washed three times with ultrapure water and lyophilized as mentioned above. The remaining weight (RW) of the scaffolds was calculated as follows:

$$\text{RW (\%)} = \frac{\text{Wt}}{\text{Wo}} \text{ x } 100, \qquad (2)$$

where $W_t$ is the dry weight of the scaffold at a predetermined time.

### 1.8 Mechanical strength analysis

*Static measurements*

Unconfined compression tests were performed in both wet and dry states. For wet state measurement, the scaffolds were previously equilibrated in cell culture medium for 3 h. The height and diameter of the samples were determined with a digital caliper gage. Samples were measured in triplicate on a Universal Tester, Instron 4204 (Norwood, USA), equipped with a static 1 kN load cell (Instron 2525 Series) and 50 mm compression platens. The samples were compressed to 40% of their initial height at a rate of 2.4 mm $min^{-1}$ and the elastic relaxation of the wet scaffolds was determined at a relaxation rate of 2.4 mm $min^{-1}$. Data analysis was performed according to the literature: Elastic modulus was determined form the initial slope of the stress-strain curve and the compressive strength equals to the compressive stress of the samples at 30% compressive strain.

**Table S1.** Detected peaks for neat Coll, citric acid, scaffolds before and after DHT and neutralization, NFC and CMC.

| Spectra | Number ($cm^{-1}$) | Peak assignment | Reference |
|---|---|---|---|
| **Coll** | | | |
| Amide A | 3303 | N-H streching vibrations | [2, 3] |
| Amide B | 2927 | C-H streching vibrations | [2, 3] |
| Amide I | 1630 | C=O streching vibrations | [2, 3] |
| Amide II | 1544 | C-N streching vibrations | [2, 3] |
| Amide III | 1238 | N-H bending, *C-N stretching and N-H in-plan bending vibration &*<br>*(sulfate groups, SO3-,asymmetric stretching vibration)* | [2, 3] |
| **Citric acid** | | | |
| | 3290 | O-H streching | [4] |
| | 1721 | C=O streching | [4] |
| | 1105 | C-OH streching | [4] |

| | | | |
|---|---|---|---|
| | 778 | $CH_2$ rocking | [4] |
| **Non DHT Coll-free and Coll scaffolds** | | | |
| | 3347 | O- H streching (and NH streching?) | [5-7] |
| | 2898 | C-H streching | [6, 8] |
| | 1712 | C=O streching | [9, 10] |
| | **1561** | **N-H bending** | [9, 10] |
| | 1394 | $COO^-$ stretch vibration | [11] |
| | 1326 | C-O streching | [9, 12] |
| | 1206 | C-O streching | |
| | 1055 | C-O-C stretching | [7, 12] |
| | 1030 | C-O streching | [2] |
| **DHT and neutralised Coll-free and Coll scaffolds** | | | |
| | 3317 | O- H streching | |
| | 2898 | C-H streching | [8] |
| | 1730 | Ester carbonyl | [13] |
| | **1577** | **Amide bond** | [14] |
| | 1398 | $COO^-$ stretch vibration - citrate shift - the formation of hydrogen bonds with collagen residues that lengthen and weaken the carboxyl groups | [15] |
| | 1326 | From CMC- OH bending | [16] |
| | 1206 | C-O streching | |
| | 1060 | C-O-C stretching | [7, 12] |
| | 1030 | C-O streching | [2] |
| **NFC** | | | |
| | 3337 | OH streching | [13] |
| | 2910 | CH streching | [13] |
| | 1100 | CO streching | [13] |
| **CMC** | | | |
| | 3298 | OH streching | [13] |
| | 2910 | CH streching | [13] |
| | 1590 | COOH streching | [13] |
| | 1100 | CO streching | [13] |

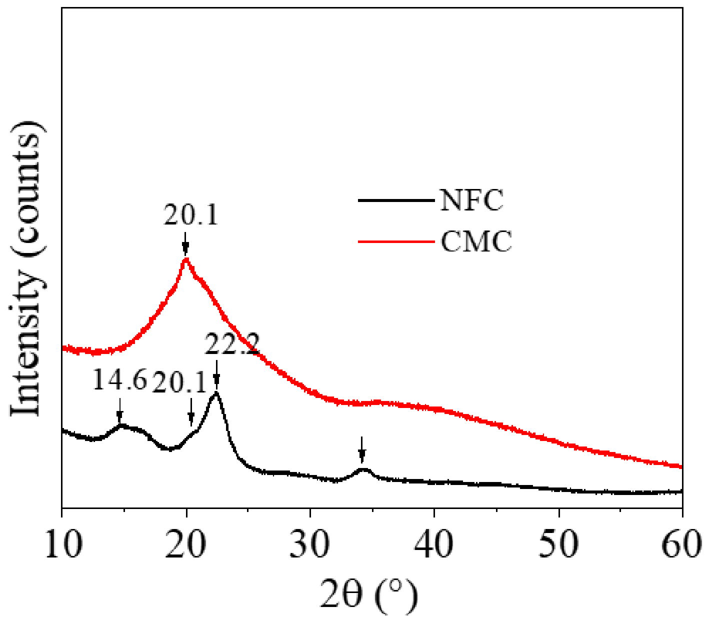


**Figure S1**. XRD spectra of neat polymers (NFC and CMC).

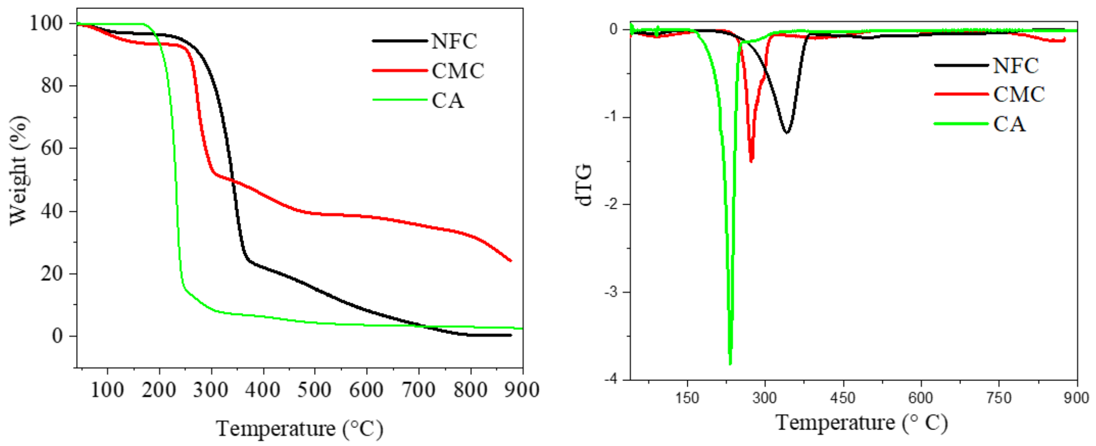


**Figure S2.** TGA (left) and dTG (right) curves of neat NFC, CMC and CA. NFC – nanofibrillated cellulose, CMC – carboxymethyl cellulose and CA-citric acid.

**NOTE (TGA; dTG).** In the first step (60–120°C), The curves show that a peak in dTG (the change in mass loss rate) occurred for both scaffold samples at 100 °C (NFC, 85 °C; CMC, 92 °C; CA, 90 °C) which can be associated with the desorption of physically bound water. After reaching a temperature of 390 °C, the mass loss for NFC is 74%. In contrast, CMC loses 46% of its mass at 390 °C, which can be attributed also to the decarboxylation and decomposition of

CMC [17]. The highest mass loss is observed for CA (mass loss = 93%), which can be attributed decomposition of CA.

## References

(1) Schindelin, J.; Arganda-Carreras, I.; Frise, E.; Kaynig, V.; Longair, M.; Pietzsch, T.; Preibisch, S.; Rueden, C.; Saalfeld, S.; Schmid, B.; et al. Fiji: an open-source platform for biological-image analysis. *Nature Methods 2012 9:7* **2012**, *9* (7), 676-682. DOI: 10.1038/nmeth.2019.
(2) Gómez Morales, J.; Fernández Penas, R.; Verdugo-Escamilla, C.; Degli Esposti, L.; Oltolina, F.; Prat, M.; Iafisco, M.; Fernández Sánchez, J. Bioinspired Mineralization of Type I Collagen Fibrils with Apatite in Presence of Citrate and Europium Ions. *Crystals* **2018**, *9* (1), 13. DOI: 10.3390/cryst9010013.
(3) Srinivasaiah, S.; Musumeci, G.; Mohan, T.; Castrogiovanni, P.; Absenger-Novak, M.; Zefferer, U.; Mostofi, S.; Bonyadi Rad, E.; Grün, N. G.; Weinberg, A. M.; et al. A 300 µm Organotypic Bone Slice Culture Model for Temporal Investigation of Endochondral Osteogenesis. *https://home.liebertpub.com/tec* **2019**, *25* (4), 197-212. DOI: 10.1089/TEN.TEC.2018.0368.
(4) Pimpang, P.; Sumang, R.; Choopun, S. Effect of Concentration of Citric Acid on Size and Optical Properties of Fluorescence Graphene Quantum Dots Prepared by Tuning Carbonization Degree. *Chiang Mai J Sci* **2018**, *45* (5), 2005-2014.
(5) Chen, X.; Zhou, L.; Xu, H.; Yamamoto, M.; Shinoda, M.; Kishimoto, M.; Tanaka, T.; Yamane, H. Effect of the Application of a Dehydrothermal Treatment on the Structure and the Mechanical Properties of Collagen Film. *Materials (Basel)* **2020**, *13* (2). DOI: 10.3390/ma13020377 From NLM.
(6) Mohan, T.; Dobaj Štiglic, A.; Beaumont, M.; Konnerth, J.; Gürer, F.; Makuc, D.; Maver, U.; Gradišnik, L.; Plavec, J.; Kargl, R.; et al. Generic Method for Designing Self-Standing and Dual Porous 3D Bioscaffolds from Cellulosic Nanomaterials for Tissue Engineering Applications. *Acs Appl Bio Mater* **2020**, *3* (2), 1197-1209. DOI: 10.1021/acsabm.9b01099 From NLM.
(7) Lohrasbi, S.; Mirzaei, E.; Karimizade, A.; Takallu, S.; Rezaei, A. Collagen/cellulose nanofiber hydrogel scaffold: physical, mechanical and cell biocompatibility properties. *Cellulose* **2020**, *27* (2), 927-940. DOI: 10.1007/s10570-019-02841-y.
(8) Bernard, F. L.; Rodrigues, D. M.; Polesso, B. B.; Chaban, V. V.; Serefin, M.; Dalla Vecchia, F.; Einloft, S. DEVELOPMENT OF INEXPENSIVE CELLULOSE-BASED SORBENTS FOR CARBON DIOXIDE. *Brazilian Journal of Chemical Engineering* **2019**, *36* (1), 511-521. DOI: 10.1590/0104-6632.20190361s20170182.
(9) Wu, X.; Liu, Y.; Liu, A.; Wang, W. Improved thermal-stability and mechanical properties of type I collagen by crosslinking with casein, keratin and soy protein isolate using transglutaminase. *Int J Biol Macromol* **2017**, *98*, 292-301. DOI: 10.1016/j.ijbiomac.2017.01.127 From NLM.
(10) Haugh, M. G.; Jaasma, M. J.; O'Brien, F. J. The effect of dehydrothermal treatment on the mechanical and structural properties of collagen-GAG scaffolds. *J Biomed Mater Res A* **2009**, *89* (2), 363-369. DOI: 10.1002/jbm.a.31955 From NLM.
(11) Shao, C.; Zhao, R.; Jiang, S.; Yao, S.; Wu, Z.; Jin, B.; Yang, Y.; Pan, H.; Tang, R. Citrate Improves Collagen Mineralization via Interface Wetting: A Physicochemical Understanding of Biomineralization Control. *Adv Mater* **2018**, *30* (8). DOI: 10.1002/adma.201704876 From NLM.
(12) Ooi, K. S.; Haszman, S.; Wong, Y. N.; Soidin, E.; Hesham, N.; Mior, M. A. A.; Tabata, Y.; Ahmad, I.; Fauzi, M. B.; Mohd Yunus, M. H. Physicochemical Characterization of Bilayer Hybrid Nanocellulose-Collagen as a Potential Wound Dressing. *Materials* **2020**, *13* (19), 4352.
(13) Lackner, F.; Liu, H.; Stiglic, A. D.; Bracic, M.; Kargl, R.; Nidetzky, B.; Mohan, T.; Kleinschek, K. S. 3D Printed Porous Nanocellulose-Based Scaffolds As Carriers for Immobilization of Glycosyltransferases. *Acs Appl Bio Mater* **2022**, *5* (12), 5728-5740. DOI: 10.1021/acsabm.2c00763.
(14) Dobaj Štiglic, A.; Kargl, R.; Beaumont, M.; Strauss, C.; Makuc, D.; Egger, D.; Plavec, J.; Rojas, O. J.; Stana Kleinschek, K.; Mohan, T. Influence of Charge and Heat on the Mechanical Properties of Scaffolds

from Ionic Complexation of Chitosan and Carboxymethyl Cellulose. *ACS Biomater Sci Eng* **2021**, *7* (8), 3618-3632. DOI: 10.1021/acsbiomaterials.1c00534 From NLM.
(15) C, S.; R, Z.; S, J.; S, Y.; Z, W.; B, J.; Y, Y.; H, P.; R, T. Citrate Improves Collagen Mineralization via Interface Wetting: A Physicochemical Understanding of Biomineralization Control. *Advanced materials (Deerfield Beach, Fla.)* **2018**, *30* (8). DOI: 10.1002/ADMA.201704876.
(16) Cukrowicz, S.; Grabowska, B.; Kaczmarska, K.; Bobrowski, A.; Sitarz, M.; Tyliszczak, B. Structural Studies (FTIR, XRD) of Sodium Carboxymethyl Cellulose Modified Bentonite. *Arch Foundry Eng* **2020**, *20* (3), 119-125. DOI: 10.24425/afe.2020.133340.
(17) Xing, R.; Wang, X.; Zhang, C.; Wang, J.; Zhang, Y.; Song, Y.; Guo, Z. Superparamagnetic magnetite nanocrystal clusters as potential magnetic carriers for the delivery of platinum anticancer drugs. *Journal of Materials Chemistry* **2011**, *21* (30), 11142-11149. DOI: 10.1039/C1JM11369K.
